# Bullying experiences in childhood and health outcomes in adulthood

**DOI:** 10.1371/journal.pone.0305005

**Published:** 2024-07-15

**Authors:** Yurie Momose, Hiroshi Ishida

**Affiliations:** Institute of Social Science, University of Tokyo, Tokyo, Japan; Universidad Santiago de Cali, COLOMBIA

## Abstract

This study examines whether the experience of being bullied at school has a long-term impact on three health outcomes in adulthood in Japan: subjective health, mental health, and activity restriction due to health conditions. We employed a random effects model and the Karlson-Holm-Breen method to decompose the total effect of being bullied at school on health inequality into a direct effect and an indirect effect working through intervening factors including education, marriage, economic well-being, and social networks. We used the Japanese Life Course Panel Surveys 2007–2020 (waves 1–14), a nationally representative panel data set that includes 2,260 male and 2,608 female respondents. The results demonstrate that for both men and women, the direct effect of being bullied at school was strong and significant. Bullying experiences in childhood had a long-term impact on health outcomes in adulthood, regardless of social background and mediating factors of education, marriage, economic well-being, and social networks. Bullying victimization increased the risk of poor subjective health, low mental health scores, and activity restriction due to health conditions. Intervening factors (especially economic well-being and friendship) mediated the association between bullying experiences and all health outcomes, but their contributions were modest. Policy measures not only to prevent bullying during childhood but also to alleviate its negative consequences in adulthood should be considered to help people who have encountered adverse childhood experiences.

## 1. Introduction

### 1.1 Bullying and health inequalities

Adverse childhood experiences (ACEs) refer to a set of negative events that individuals encounter during childhood and are known to be associated with health inequality. A large body of research documents the association between ACEs and the risk of negative health consequences [1–6]. Traditionally, the focus of research on childhood trauma and negative events has been on acute or exceptionally high-stress events such as parental death and abuse [7–10]. In recent years, among a variety of ACEs, being the victim of bullying in childhood has received increasing attention [11–14]. It is widely recognized that bullying experiences can have a wide range of effects. For example, being a victim of bullying has an impact on the well-being of children, including subjective health, life satisfaction, anxiety disorders, depression, physical symptoms, sleep disturbances, loneliness, social isolation, low self-esteem, feelings of hopelessness, self-harm, and aggression.

Bullying experiences at schools are recognized as a significant social concern, affecting about one in three adolescents worldwide [15]. Japanese people are believed to be less likely than Americans to commit any kind of deviant behavior [16]. However, Kobayashi and Farrington [17] reported that Japanese students tended to bully more than American students because they had more tolerant attitudes toward bullying. Similarly, Yoneyama and Naito [18] summarized the prevalence of bullying in Japan, claiming that bullying involving the entire class was one of the most common forms of bullying in Japan. According to a survey conducted by Iwanaga et al. [19], 42% of Japanese adults living in a metropolitan area, who participated in the study, reported being victims of bullying during their elementary and middle school years. These results confirm that bullying stands as one of the most significant educational and social challenges in Japan.

The experience of bullying can negatively affect social factors such as educational attainment and socio-economic status, create difficulties in building social networks due to social isolation, and result in dissatisfaction with human relationships [20–22]. Research on the association between bullying experiences and health has mainly focused on mental health [23, 24], with comparatively little evidence about the effects on individuals’ physical health [14, 25, 26]. Even though an increasing number of studies reveal long-lasting impacts of bullying victimization, the effects appear to be mixed, and the mechanisms are not readily apparent [22].

This growing attention to bullying experiences is related to a surge in research examining the physical and mental health effects of trauma and negative events over the past 50 years [27]. Priest et al. [28] found that the combined and cumulative experiences of bullying victimization and racism exacerbate the risks to mental and physical health. Wolke and Lereya [22, p.879] contend that “being bullied is still often wrongly considered as a ‘normal rite of passage’” and that childhood bullying has been ignored as a major public health concern. Due to these findings, the importance of examining the link between bullying and health status has increased over the years, extending even to researchers focusing on health inequalities.

### 1.2 Long-term effects of bullying in adulthood

In recent years, there has been growing concern that bullying victimization can have negative consequences not only during childhood but also throughout one’s life. According to a review by Arseneault [29], the effects of being bullied may persist even after the bullying has stopped. Smokowski and Kopasz [30] summarized research on bullying and concluded that victims of bullying in childhood suffer long-term effects on adult mental health, including anxiety, depression, substance use, and behavioral disorders. They suggested that addressing bullying can not only reduce mental health symptoms in children, but also prevent mental health problems in adulthood. Their review further argued that bullying should be seen as another form of childhood abuse, along with parental physical abuse and neglect. The experience of being bullied can lead to the worst possible consequences later in life, such as victims harming the lives of others by carrying a weapon [31], or engaging in interpersonal violence [32], or taking their own lives [33–35].

Recent research has expanded the investigation of the effects of ACEs on health disparities to include the elderly population. In this context, Hu [36] explored this topic, focusing on the consequences of childhood bullying experiences on the mental well-being and life satisfaction of Chinese individuals aged 60 years and above. The study emphasized that the detrimental effects of bullying victimization on depressive symptoms persist even after adjusting for confounding variables. However, it noted a potential reduction of these effects among the very elderly. Both Guo et al. [37] and Chen et al. [38] incorporated a comprehensive array of ACE factors, such as parental divorce, parental death, household mental illness, domestic violence, and bullying, among others, to assess their collective influence on the mental health of the elderly. Meanwhile, Li et al. [39] showcased how ACEs affect the activities of daily living among older adults.

Most of the prior research on ACEs typically amalgamated various adverse experiences, including bullying victimization, into a cumulative score to measure the overall extent of ACEs. This approach poses challenges in isolating the specific influence of bullying experiences. An exception to this trend is the study by Zhou and Zhou [40], which delineated the individual components of ACEs and explored the direct and indirect effects of childhood bullying experiences on mental health. Our study centers on childhood bullying victimization, assessing its influence not only on mental well-being but also on physical health outcomes in adulthood. We aim to investigate whether the pathways linking childhood bullying experiences to adult health outcomes differ depending on whether mental or physical health is affected.

In addition, many studies have incorporated gender differences into their analytical frameworks, given that prior research on bullying encouraged the inclusion of gender as a factor in the analysis [41]. Cao et al. [42], having studied the impact of bullying on mental health, broke down the effects of bullying on suicidal ideation into direct effects and indirect effects mediated by loneliness, finding that women were more likely to suffer from psychological maladjustment after being victims of bullying. Other studies found that women are more likely to experience the detrimental effects of frequent bullying and have a higher incidence of depression [33, 43, 44]. In contrast, there are findings that indicate that men are more likely to be victims of bullying than women because they are more likely to be rejected by their peers, and that men also have poorer physical health due to bullying [45–47]. Furthermore, while boys are more likely to be the target of physical and verbal bullying, girls are more likely to encounter cyberbullying and relationship exclusion [48]. These studies suggest that neither women nor men have a monopoly on the suffering that bullying can induce, nor is it certain that one gender consistently encounters more severe consequences as a result of being bullied.

In summary, research on the health effects of bullying victimization at school has gained momentum in recent years, indicating emerging evidence of long-term physical and psychological consequences. However, the mechanisms behind the long-lasting effects of bullying experiences in childhood on health inequality in adulthood require further investigation. As stated by Shaw et al. [48], the negative effects of traditional and cyberbullying are well established, but it is not clear whether they result from the direct effect of bullying or from indirect effects via mediating factors in adulthood.

### 1.3 Contribution of our research

The present study aims to make three significant contributions. First, it reveals that the experience of being bullied at school during childhood yields enduring negative consequences that extend into adulthood in Japan. By focusing on the long-term ramifications of bullying, this study transcends the confines of school environments, shedding light on the need for interventions and support mechanisms for individuals who have endured past victimization. Previous studies [29, 30] have emphasized the necessity of further understanding the mechanisms underlying the persistent negative outcomes of bullying. Drawing from positive psychology, which often explores social behavior, psychological resilience, and life satisfaction as avenues for preventing bullying in schools [49], this study extends beyond the school setting to provide insights into the post-school experiences of adult victims of bullying, enriching our understanding of their journey into adulthood.

Second, this study examines three key health outcomes, encompassing not only mental health but also subjective health and activity limitations. Whereas previous research on bullying and health inequality has predominantly centered on mental health [23, 24], our study broadens the scope to address a wider spectrum of health disparities. In the realm of health, inequalities extend beyond mental well-being to cover subjective health perceptions and limitations in daily activities due to health issues. By considering these multifaceted health dimensions, this research contributes to a more comprehensive understanding of the impact of school bullying on individuals’ overall well-being.

Third, the study explores pathways linking childhood bullying to adult health by investigating both direct and indirect mechanisms through which bullying influences adult health. Apart from Zhou and Zhou [40], to the best of our knowledge, previous research has not decomposed the long-term impact of bullying on adult health into direct and indirect pathways. Zhou and Zhou [40] offered breakdowns of ACEs into components (including bullying experiences) and examined their specific effects. They found that the predominant path from childhood bullying experiences to adult mental health is direct (80%). Our research utilizes this finding as a benchmark.

However, there are notable differences between Zhou and Zhou’s study and our research: (1) While Zhou and Zhou examined the impact of adverse childhood experiences on mental health, our study explores both mental and physical health outcomes; (2) Zhou and Zhou utilized subjective variables, such as satisfaction with children and partners, social activities, and social support, as mediators, whereas our research incorporates objective life-course events, such as educational attainment, economic status, marriage, and the presence of social network ties. The differences in mediating factors between the two studies may lead to varying decomposition results. We posit that our study represents one of the first systematic examinations of the role of objective life-course events in mediating the association between childhood bullying experiences and adult health outcomes. By comprehending the diverse mediating pathways through life-course events, this research provides valuable insights for shaping future interventions and support mechanisms for individuals with a history of bullying victimization.

## 2. Methods

### 2.1 Data

#### Data description

The data set used in this study comes from the Japanese Life Course Panel Surveys (JLPS) 2007–2020 (waves 1 through 14), a nationally representative panel data set, conducted every year since 2007 (the continuous sample). The JLPS is composed of the youth sample (respondents aged 20 to 34 years in 2007) and the middle-aged sample (respondents aged 35 to 40 years in 2007). The JLPS added the refresh youth sample in 2019 in order to compensate for the aging of the original sample respondents. The refresh youth sample includes respondents who were aged 20 to 31 years in 2019. After deleting cases with missing values, 2,260 male respondents and 2,608 female respondents were used for the analyses. For further details regarding the JLPS, see Ishida [50, 51] and Ishida, Arita, and Fujihara [52].

#### Sampling procedure

The continuous sample and the refresh sample used an identical sampling procedure. Primary sampling units were selected following a process of stratification based on geographical regions (10 regions: Hokkaido, Tohoku, Kanto, Hokuriku, Tozan, Tokai, Kinki, Chugoku, Shikoku, and Kyushu) and urban classification (four categories: 16 largest cities, cities with populations exceeding 200 million, other cities, and towns/villages). From each sampling unit, respondents were selected using the Basic Resident Register and Electoral Register, which provided comprehensive lists of all residents. Before individual respondent selection, stratification by age (20–24, 25–29, 30–34, and 35–40) and gender (male and female) was conducted, ensuring adequate representation from each age and gender group. The respondents constituted a nationally representative sample of men and women across the targeted age groups [53].

The continuous sample yielded valid responses from 3,367 individuals for the youth sample and 1,433 individuals for the middle-aged sample. The response rates were 34.5% for the youth sample and 40.4% for the middle-aged sample. The refresh youth sample collected valid responses from 2,051 individuals, achieving a response rate of 35.6%. These respondents have been followed up annually, with retention rates of approximately 80% for the youth sample (for both the continuous and refreshed samples) and approximately 88% for the middle-aged sample.

#### Informed consent and IRB approval

Respondents received an informed consent form by mail in January 2007 (for the continuous sample) and January 2019 (for the refresh sample), describing the study’s objectives and purpose, along with details regarding the confidentiality and anonymity of responses. This information was provided prior to the distribution of the questionnaires. Subsequently, the questionnaires were dispatched by mail to all participants, excluding those who opted out of the survey by responding negatively to the initial mail. Trained surveyors from a reputable survey firm conducted visits to the respondents and collected the completed questionnaires. In the case of the 2007 continuous sample, verbal consents were obtained, and these were duly documented by the surveyors at the time of collecting the completed questionnaires. For the 2019 refresh sample, written consents were procured and collected by trained surveyors. Respondents who did not provide consent were excluded from the sample.

The Institutional Research Ethics Review Board (the Research Ethics Review Committee) at the Institute of Social Science, The University of Tokyo, approved the Japanese Life Course Panel Surveys (JLPS) project. We declare compliance with the ethical practices described in the Code of Conduct for Research at The University of Tokyo.

### 2.2 Variables

**Fig 1:**
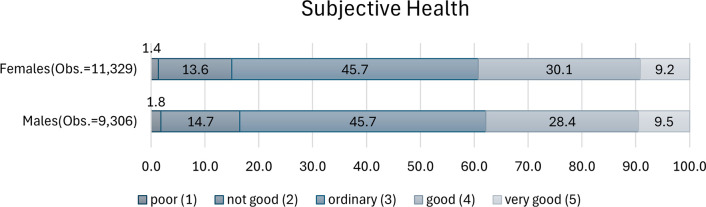
Frequency distribution of subjective health.

We used three outcome variables: subjective health, mental health score, and no activity restriction. Subjective health was measured using the following question: “How do you feel about your general health?” This question does not specify whether it pertains to physical or mental health; it is intended to inquire about the respondent’s overall general health. Responses were coded as: “poor” (1), “not good” (2), “ordinary” (3), “good” (4), and “very good” (5). Fig 1 shows the distribution by gender, revealing that less than 20% of respondents reported poor or not good health, 46% ordinary health, and the remainder good or very good health. The distributions exhibit a near-identical pattern between male and female respondents.

**Fig 2:**
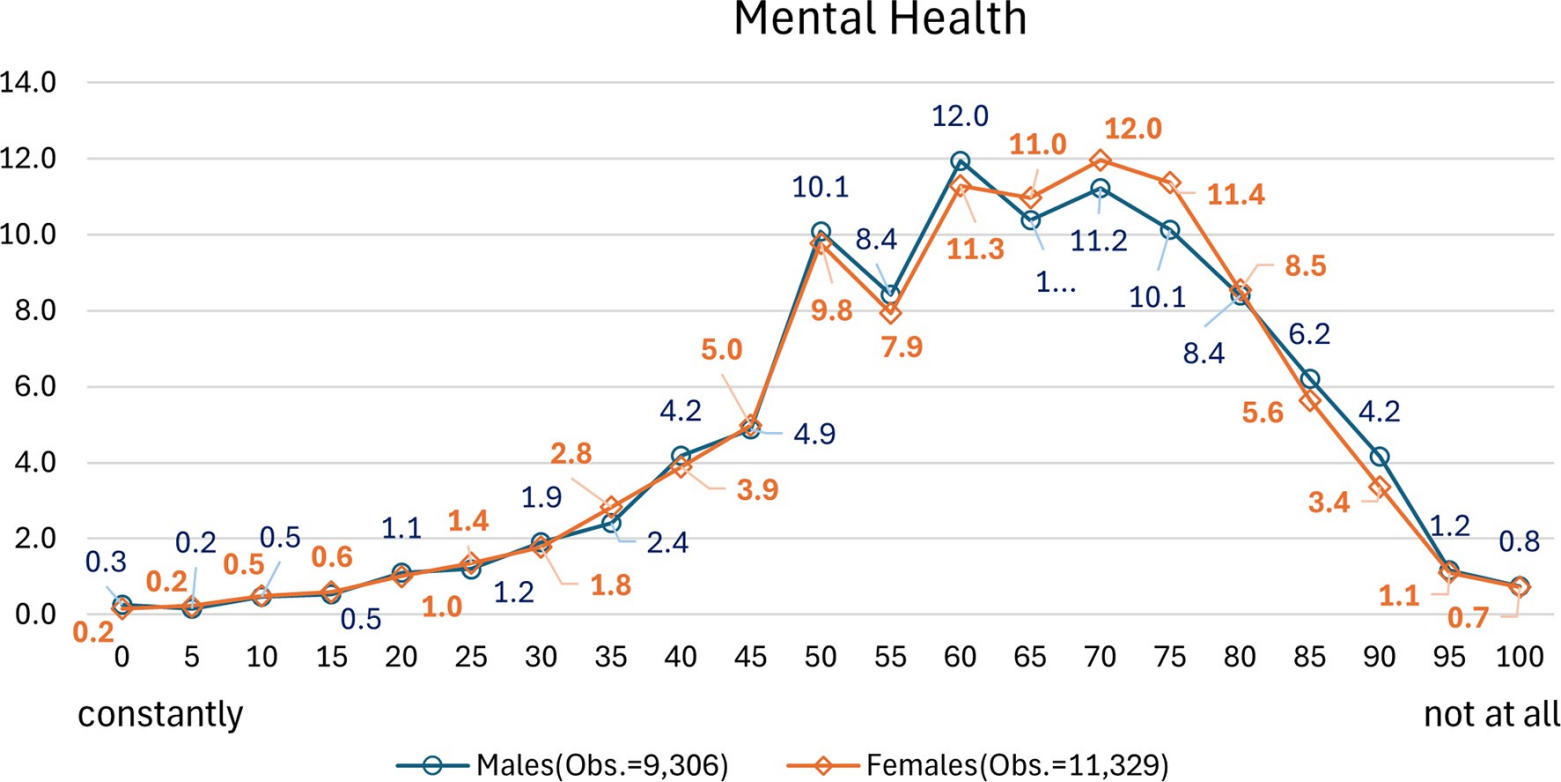
Frequency distribution of mental health.

Mental health scores were computed by summing the responses to five questions about the respondent’s mental state: “How often in the last month did you feel the following? A) Feeling quite nervous, B) Feeling so down in the dumps that nothing could cheer you up, C) Feeling calm and peaceful, D) Feeling downhearted and blue, and E) Feeling happy.” Responses were coded as “constantly” (1), “nearly constantly” (2), “occasionally” (3), “rarely” (4), and “not at all” (5). For items C and E, the response categories were reversed so that the higher the value, the better the mental state. The coefficient of reliability for these five items was 0.788 (Cronbach’s alpha), indicating an acceptable level of reliability. Fig 2 displays the distribution of mental health scores by gender. These show remarkable similarity up to a score of 50, after which point female respondents tend to exhibit slightly higher scores, indicating a better mental state than male respondents.

**Fig 3:**
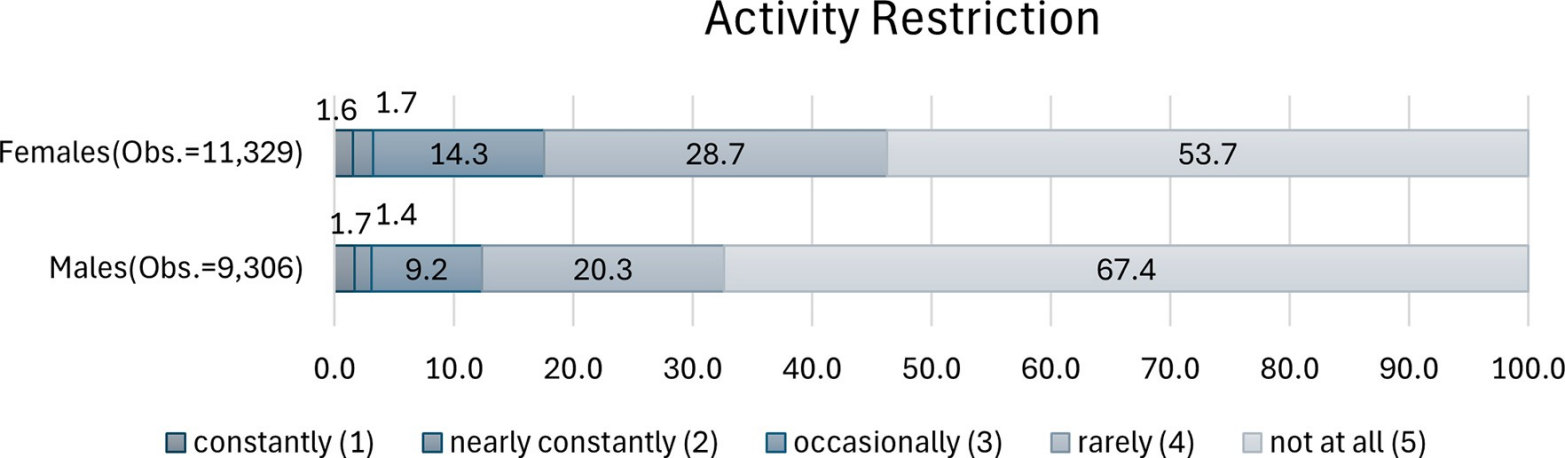
Frequency distribution of activity restriction.

The variable “no activity restriction” was coded using the question: “How often in the last month did you feel that activities like housekeeping and work were limited because of your health conditions?” Responses with “rarely” and “not at all” were coded as 1, and 0 otherwise. Fig 3 describes the distributions of activity restriction by gender. The figure clearly demonstrates that male respondents were more inclined to respond with “not at all” compared to female respondents, suggesting that men reported experiencing less restriction in their daily activities due to health issues than women.

The treatment variable is the experience of bullying at school. Among the list of events which the respondents experienced, those who reported yes to the item, “I was bullied at school,” were coded 1, and 0 otherwise. A number of social background variables were included as controls. Father’s and mother’s educations were coded as 1 when they attended two-year junior college or four-year university and 0 otherwise. An indicator variable for “unknown” was included due to a relatively large number of cases with missing responses. The number of books at home when the respondent was 15 years old was used as an indicator of household cultural capital. We created two measures to capture economic well-being when the respondent was growing up. The first variable is a binary indicator signifying whether the respondent was living in a home owned by their parents when he/she was 15 years old. Second, a question about the standard of living when the respondent was 15 years old was coded as “wealthy” (5), “somewhat wealthy” (4), “average” (3), “somewhat poor” (2), and “poor” (1). Finally, a variable indicating whether the respondent had any life-restricting illness or disorder before he/she was 18 years old was created to measure baseline health conditions. These background variables are time-invariant and fixed for the entire period of the panel survey.

We also introduced a number of mediating variables. The respondent’s educational attainment was coded as 1 when the respondent attended institutions of higher education and 0 otherwise. This variable is time-invariant and did not change across waves. The following mediating variables are time-variant and may change across waves. Being unmarried was determined by a marital status question asked in every wave. Current living standard was measured by the self-reported standard of living in each wave: “wealthy” (5), “somewhat wealthy” (4), “average” (3), “somewhat poor” (2), and “poor” (1). Whether the respondent had a friend in every wave was used to determine the “currently has no friends” variable. Four network-related questions were asked to determine the extent of the respondent’s social network ties: “Who do you talk to when you want to discuss the following matters? A) about work or study, B) about job referrals, C) about relationships with friends and partners, D) about borrowing significant amounts of money when you are in need because of job loss or illness.” A list of possible people (such as parents, partners, children, and siblings) that the respondent may specify was provided. If the respondent replied “none,” the variable is coded as 1, and 0 when the respondent chose any one of the listed people. These four social network variables were labelled as follows: “no one to talk to about work and study,” “no one to talk to about job referral,” “no one to talk to about relationships,” and “no one to talk to about borrowing money.” These questions were asked every other wave.

Finally, age and age squared were included as controls. We also controlled for survey samples (youth sample, middle-aged sample, and refresh youth sample), and the waves (wave 1 to wave 14) but do not show these coefficients in the following tables. Descriptive statistics are shown in Table 1.

**Table 1:** Descriptive statistics.

	All (Obs. = 20,635)					Males (Obs. = 9,306)					Females (Obs. = 11,329)				
	Mean	Std. Dev.	Min	Max	Median	Mean	Std. Dev.	Min	Max	Median	Mean	Std. Dev.	Min	Max	Median
**Three Health Outcomes**															
Subjective Health	2.307	0.880	0	4	2	2.290	0.893	0	4	2	2.321	0.869	0	4	2
Mental Health	62.55	17.43	0	100	65	62.59	17.54	0	100	65	62.52	17.33	0	100	65
No Activity Restriction	0.848	0.359	0	1	1	0.877	0.329	0	1	1	0.825	0.380	0	1	1
**Treatment variable**															
Bullying at school	0.228	0.420	0	1	0	0.188	0.391	0	1	0	0.261	0.439	0	1	0
**Mediating variables**															
Respondent’s higher education	0.521	0.500	0	1	1	0.532	0.499	0	1	1	0.512	0.500	0	1	1
Currently unmarried	0.398	0.490	0	1	0	0.438	0.496	0	1	0	0.366	0.482	0	1	0
Currently has no friends	0.024	0.154	0	1	0	0.037	0.188	0	1	0	0.014	0.118	0	1	0
Current living standard	2.027	0.773	0	4	2	2.026	0.806	0	4	2	2.029	0.746	0	4	2
Social network items															
No one to talk to about work and study	0.063	0.243	0	1	0	0.096	0.294	0	1	0	0.036	0.187	0	1	0
No one to talk to about job referral	0.401	0.490	0	1	0	0.378	0.485	0	1	0	0.421	0.494	0	1	0
No one to talk to about relationships	0.113	0.317	0	1	0	0.186	0.389	0	1	0	0.054	0.225	0	1	0
No one to talk to about borrowing money	0.151	0.358	0	1	0	0.186	0.389	0	1	0	0.123	0.328	0	1	0
**Social background variables**															
Father’s education															
No higher education	0.598	0.490	0	1	1	0.586	0.493	0	1	1	0.608	0.488	0	1	1
Higher education	0.276	0.447	0	1	0	0.282	0.450	0	1	0	0.271	0.444	0	1	0
Unknown	0.126	0.332	0	1	0	0.132	0.338	0	1	0	0.122	0.327	0	1	0
Mother’s education															
No higher education	0.708	0.455	0	1	1	0.681	0.466	0	1	1	0.730	0.444	0	1	1
Higher education	0.177	0.382	0	1	0	0.178	0.382	0	1	0	0.177	0.382	0	1	0
Unknown	0.115	0.319	0	1	0	0.141	0.348	0	1	0	0.093	0.290	0	1	0
Number of books at home when 15 years old	87.90	110.76	0	500	38	91.86	117.24	0	500	38	84.65	105.02	0	500	75
Homeownership when 15 years old	0.785	0.411	0	1	1	0.770	0.421	0	1	1	0.796	0.403	0	1	1
Living standard when 15 years old	2.074	0.810	0	4	2	2.060	0.811	0	4	2	2.086	0.809	0	4	2
Illness or disorder before 18 years old	0.037	0.190	0	1	0	0.037	0.190	0	1	0	0.037	0.190	0	1	0
Age	36.014	7.331	20	52	36	35.864	7.205	20	52	36	36.137	7.431	20	52	36
Age squared	1350.75	526.42	400	2704	1,296	1338.15	514.84	400	2704	1,296	1361.10	535.55	400	2704	1,296
**Survey samples**															
Youth sample	0.636	0.481	0	1	0	0.655	0.475	0	1	0	0.620	0.486	0	1	0
Middle-aged	0.313	0.464	0	1	1	0.298	0.457	0	1	1	0.325	0.469	0	1	1
Refresh youth sample	0.051	0.221	0	1	0	0.047	0.212	0	1	0	0.055	0.228	0	1	0
Male	0.451	0.498	0	1	0	1.000	0.000	1	1	1	0.000	0.000	0	0	0

### 2.3 Statistical model

To take advantage of the panel-type data set, we use a random effects model. This method can include both time-invariant predictors and time-varying predictors and can be written as follows:

$$y_{it}=\theta_{t}+\gamma z_{i}+\beta x_{it}+\alpha_{i}+\epsilon_{it}$$ (1)

where y_it_ is the outcome variable, *θ_t_* is a set of time indicator variables, z_i_ is a set of time-invariant predictors, and x_it_ is a set of time-varying predictors. α_i_ is a set of random variables with a specified probability distribution and is assumed to be independent of the rest of the variables in the equation. Finally, ϵ_it_ is an error term.

In order to decompose the effect of the treatment (bullying experiences) on the outcome, we use the Karlson-Holm-Breen (KHB) method [54–57]. We aim to estimate the share of the direct and indirect pathways in the total effect. However, a comparison of the total and direct effects of the treatment on the outcome in nested nonlinear models is affected by both confounding due to mediating variables and rescaling of the model. In other words, when a mediating variable is included in a nonlinear equation model, the coefficient of the treatment variable can change, regardless of whether it is correlated with that mediator, as long as the mediator is correlated with the outcome. To address different scaling across models, we employ the KHB method. This method not only estimates the effect of confounding (the indirect portion) net of rescaling but also provides a statistical test for the indirect effect. Other decomposition methods often lack a statistical test for indirect component [58]. Because our analyses include both linear and nonlinear equations, the KHB method emerges as the most suitable for decomposition [59]. This method has been used extensively not only in the sociological literature but also in health sciences [37, 38, 40].

## 3. Results

Tables 2–4 shows the results of fitting two models by gender separately for three health outcomes. The first model predicts health outcomes by the treatment variable (bullying at school) and confounding variables (social backgrounds). The second model predicts health outcomes by bullying at school, social background variables, and mediating variables.

**Table 2:** Random effect models of predicting subjective health by gender.

	Subjective Health											
	Males						Females					
	Model 1			Model 2			Model 1			Model 2		
	*Coef*.		*Std*. *Err*.	*Coef*.		*Std*. *Err*.	*Coef*.		*Std*. *Err*.	*Coef*.		*Std*. *Err*.
**Treatment variable**												
Bullying at school	-0.153	**	0.039	-0.114	**	0.038	-0.173	**	0.031	-0.153	**	0.029
**Mediating variables**												
Respondent’s higher education				0.051		0.032				0.085	**	0.028
Currently unmarried				-0.068	**	0.025				-0.094	**	0.022
Currently has no friends				-0.160	**	0.048				-0.163	*	0.067
Current living standard				0.177	**	0.012				0.134	**	0.011
Social network items												
No one to talk to about work and study				-0.042		0.030				-0.113	**	0.042
No one to talk to about job referral				-0.073	**	0.019				-0.014		0.016
No one to talk to about relationships				-0.043		0.024				-0.100	**	0.036
No one to talk to about borrowing money				-0.004		0.023				-0.031		0.024
**Social background variables**												
Father’s education (base: no higher education)												
Higher education	0.089	*	0.041	0.063		0.040	0.032		0.036	0.002		0.035
Unknown	-0.005		0.071	0.003		0.067	-0.078		0.060	-0.060		0.057
Mother’s education (base: no higher education)												
Higher education	0.059		0.047	0.044		0.044	0.025		0.040	0.005		0.039
Unknown	-0.063		0.068	-0.033		0.065	-0.030		0.066	-0.029		0.063
Number of books at home when 15 years old	0.000		0.000	0.000		0.000	0.000		0.000	0.000		0.000
Homeownership when 15 years old	-0.006		0.038	-0.024		0.036	0.025		0.034	0.017		0.033
Living standard when 15 years old	0.112	**	0.020	0.052	**	0.019	0.123	**	0.017	0.080	**	0.017
Illness or disorder before 18 years old	-0.351	**	0.077	-0.321	**	0.073	-0.373	**	0.068	-0.327	**	0.065
Age	-0.025	*	0.010	-0.025	*	0.010	-0.004		0.009	-0.014		0.009
Age squared	0.000		0.000	0.000		0.000	0.000	**	0.000	0.000		0.000
Constant	2.917	**	0.186	2.767	**	0.196	2.605	**	0.164	2.627	**	0.179
Number of observations	9,306						11,329					
Number of individuals	2,260						2,608					
Wald chi2	368.6			685.7			514.2			756.3		

**p <.01

*p <.05.

**Table 3:** Random effect models of predicting mental health by gender.

	Mental Health											
	Males						Females					
	Model 1			Model 2			Model 1			Model 2		
	*Coef*.		*Std*. *Err*.	*Coef*.		*Std*. *Err*.	*Coef*.		*Std*. *Err*.	*Coef*.		*Std*. *Err*.
**Treatment variable**												
Bullying at school	-6.710	**	0.770	-5.609	**	0.742	-4.503	**	0.630	-3.981	**	0.601
**Mediating variables**												
Respondent’s higher education				0.344		0.627				0.911		0.567
Currently unmarried				-2.536	**	0.488				-3.563	**	0.439
Currently has no friends				-4.120	**	0.938				-2.445		1.306
Current living standard				2.638	**	0.239				2.644	**	0.223
Social network items												
No one to talk to about work and study				-1.046		0.599				-2.547	**	0.808
No one to talk to about job referral				-1.682	**	0.365				-0.961	**	0.309
No one to talk to about relationships				-0.815		0.470				-3.097	**	0.692
No one to talk to about borrowing money				-1.756	**	0.449				-2.278	**	0.472
**Social background variables**												
Father’s education (base: no higher education)												
Higher education	2.087	**	0.805	1.731	*	0.782	0.076		0.747	-0.300		0.720
Unknown	2.527		1.376	2.835	*	1.315	-0.620		1.223	-0.203		1.170
Mother’s education (base: no higher education)												
Higher education	-0.668		0.907	-0.835		0.870	0.912		0.832	0.630		0.798
Unknown	-1.806		1.324	-1.388		1.268	-0.838		1.355	-0.914		1.293
Number of books at home when 15 years old	0.001		0.003	0.001		0.003	0.003		0.003	0.004		0.003
Homeownership when 15 years old	-0.003		0.731	-0.456		0.702	0.781		0.709	0.542		0.676
Living standard when 15 years old	1.374	**	0.388	0.401		0.380	1.710	**	0.355	0.799	*	0.345
Illness or disorder before 18 years old	-2.370		1.503	-1.732		1.440	-6.361	**	1.398	-5.178	**	1.335
Age	0.251		0.196	0.060		0.200	0.580	**	0.170	0.140		0.179
Age squared	-0.004		0.003	-0.002		0.003	-0.008	**	0.002	-0.002		0.002
Constant	56.485	**	3.663	59.539	**	3.868	49.030	**	3.226	55.640	**	3.503
Number of observations	9,306						11,329					
Number of individuals	2,260						2,608					
Wald chi2	117.6			414.3			158.3			530.8		

**p <.01

*p <.05.

**Table 4:** Random effect models of predicting no activity restriction by gender.

	No Activity Restriction											
	Males						Females					
	Model 1			Model 2			Model 1			Model 2		
	*Coef*.		*Std*. *Err*.	*Coef*.		*Std*. *Err*.	*Coef*.		*Std*. *Err*.	*Coef*.		*Std*. *Err*.
**Treatment variable**												
Bullying at school	-0.652	**	0.127	-0.555	**	0.124	-0.476	**	0.092	-0.483	**	0.093
**Mediating variables**												
Respondent’s higher education				0.127		0.110				0.159		0.090
Currently unmarried				-0.175		0.105				0.578	**	0.088
Currently has no friends				-0.407	*	0.198				-0.460		0.257
Current living standard				0.329	**	0.055				0.176	**	0.047
Social network items												
No one to talk to about work and study				-0.060		0.147				-0.267		0.176
No one to talk to about job referral				-0.038		0.091				0.036		0.068
No one to talk to about relationships				0.015		0.117				-0.152		0.149
No one to talk to about borrowing money				-0.132		0.107				-0.095		0.101
**Social background variables**												
Father’s education (base: no higher education)												
Higher education	-0.110		0.142	-0.157		0.139	-0.325	**	0.112	-0.369	**	0.114
Unknown	-0.335		0.239	-0.317		0.231	-0.211		0.187	-0.185		0.188
Mother’s education (base: no higher education)												
Higher education	0.277		0.163	0.237		0.158	0.050		0.127	-0.014		0.129
Unknown	0.327		0.232	0.377		0.226	-0.026		0.208	-0.006		0.208
Number of books at home when 15 years old	0.000		0.000	0.000		0.000	0.000		0.000	0.000		0.000
Homeownership when 15 years old	-0.013		0.128	-0.067		0.124	0.082		0.106	0.066		0.106
Living standard when 15 years old	0.034		0.068	-0.073		0.068	0.097		0.053	0.046		0.055
Illness or disorder before 18 years old	-0.802	**	0.240	-0.726	**	0.232	-1.231	**	0.193	-1.227	**	0.194
Age	-0.027		0.050	-0.035		0.051	-0.119	**	0.039	-0.033		0.041
Age squared	0.000		0.001	0.000		0.001	0.001	**	0.001	0.000		0.001
Constant	3.491	**	0.907	3.285	**	0.943	4.347	**	0.707	2.124	**	0.764
Number of observations	9,306						11,329					
Number of individuals	2,260						2,608					
Wald chi2	51.5			110.2			111.1			175.6		

**p <.01

*p <.05.

We begin with the first health outcome, subjective health, in Table 2. Let us focus on the first row, the coefficients for the school bullying variable. For both male and female respondents, the effect of bullying at school is negative and significant after controlling for social background (Model 1). The experience of being bullied during childhood is associated with worse subjective health in adulthood. After introducing mediating variables, the effect of bullying at school is reduced slightly for men and women (Model 2). However, the effect of bullying experiences continues to be substantial and statistically significant. The experience of school bullying affects subjective health regardless of education, marriage, economic well-being, and social network.

We next report the results for the second health outcome, mental health (Table 3). Just like the results for subjective health, for both men and women the effect of bullying experiences at school is negative and significant after social background variables are controlled. After introducing mediating variables, the effect of bullying experiences is reduced but remains substantial and statistically significant. The experience of being bullied during childhood is associated with a worse mental health state in adulthood, even after social background and mediating experiences of education, marriage, economic status, and social network are controlled for.

Finally, we show the results for the third health outcome, no activity restriction (Table 4). For both men and women, the school bullying variable exerts a negative and significant effect on no activity restriction after controlling for social background, and further after controlling for mediating variables. The introduction of mediating variables reduces the effect of school bullying for male respondents, but hardly changes the effect for female respondents. These results indicate that the experience of school bullying reduces the odds of having no activity restriction in adulthood, even after accounting for all confounding and mediating factors. In other words, people who had childhood experiences of bullying are more likely to experience activity restriction due to health conditions.

The findings in Tables 2–4 emphasize that negative influences associated with childhood bullying experiences at school transcend the school environment and endure well into adulthood. These consequences manifest across various domains, encompassing mental health, subjective health assessments, and constraints in daily activities attributable to health concerns.

Table 5 reports the results of the decomposition of the effect of school bullying using the KHB method. There are three panels for three health outcomes: subjective health, mental health, and no activity restriction. The row labelled “Reduced” indicates the total effect of bullying at school after social background variables are controlled, “Full” indicates the direct effect of bullying after mediating factors are introduced, and “Difference” indicates the indirect effect of bullying through mediating factors. The Reduced effects are slightly different from the effects reported in Tables 2–4 because they adjust for re-scaling. The Full effects are the same as those reported in Tables 2–4.

**Table 5:** Decomposition of the effect of school bullying on three health outcomes using the KHB method.

	Subjective Health						Mental Health						No Activity Restriction					
	Males			Females			Males			Females			Males			Females		
	*Coef*.		*Std*. *Err*.	*Coef*.		*Std*. *Err*.	*Coef*.		*Std*. *Err*.	*Coef*.		*Std*. *Err*.	*Coef*.		*Std*. *Err*.	*Coef*.		*Std*. *Err*.
Bullying at school																		
Reduced	-0.153	**	0.039	-0.173	**	0.031	-6.71	**	0.77	-4.503	**	0.63	-0.636	**	0.123	-0.474	**	0.092
Full	-0.114	**	0.038	-0.153	**	0.029	-5.609	**	0.742	-3.981	**	0.601	-0.555	**	0.124	-0.483	**	0.093
proportion of Full to Reduced	0.745			0.889			0.836			0.884			0.873			1.019		
Difference	-0.039	**	0.007	-0.019	**	0.004	-1.101	**	0.134	-0.522	**	0.082	-0.081	**	0.021	0.009		0.01
proportion of Differenc to Reduced	0.255			0.111			0.164			0.116			0.127			-0.019		

**p <.01

*p <.05.

The decomposition of the effect on subjective health (left-hand side panel of Table 5) reveals that the extent of indirect effect (the proportion of Difference to Reduced in Table 5) is 0.255 for males and 0.110 for females. In other words, about a quarter of the total effect of bullying at school on subjective health among men, and about one tenth among women, are explained by mediating factors. As shown in Table 2, among the mediating variables, the following exert significant impact on subjective health for both males and females: being unmarried, having no friends, and lower economic well-being. These factors partially account for why bullying experiences influences subjective health.

The middle panel of Table 5 shows the decomposition of the effect of school bullying on mental health. The indirect effect through mediators comprises 16% of the total effect of bullying for male respondents and 12% for female respondents. A modest but significant portion of the association between school bullying and mental health is thus explained by mediating factors. Among these mediators, marriage, economic well-being, and some social network variables significantly affect mental health, suggesting that they play an important role in mediating the influence of school bullying on mental health.

Finally, the right-hand side panel of Table 5 presents the decomposition of the effect of school bullying on no activity restriction. The indirect effect comprises 13% of the total effect for men and virtually none for women. Among men, economic well-being and having no friends appear to mediate the association between school bullying and no activity restriction as shown in Table 4. Among women, the impact of school bullying is entirely direct without going through mediating factors.

In summary, the results in Table 5 indicate that life-course events, including educational attainment, economic status, marriage, and the presence of social network ties, partially explain the connection between childhood bullying experiences and adult health outcomes. However, our analysis reveals that the primary pathway linking childhood bullying to adult health is direct, rather than being mediated by these life-course events.

## 4. Discussion

Our study first demonstrates that the experience of being bullied at school has enduring negative consequences for health outcomes in adulthood in Japan. The impacts of school bullying extend beyond the school environment and persist into adulthood. Second, our findings reveal that these enduring influences are not limited to mental health but also encompass subjective health perceptions and limitations in daily activities due to health issues, highlighting the multidimensional aspects of health inequalities that are at risk. Third, our research uncovers pathways from childhood experiences of school bullying to adult health inequalities. Individuals who were victims of school bullying continue to suffer from poor health outcomes in adulthood, both through their life-course experiences and through pathways independent of these events. Notably, the primary pathway remains direct, bypassing objective life-course events such as educational attainment, economic status, marriage, and the presence of social network ties.

Our analyses reveal that the experience of being bullied during childhood is associated with worse subjective health, lower mental health status, and activity restriction in adulthood for both men and women. The decomposition analysis shows that the impacts of school bullying are primarily direct: the experience of school bullying continues to affect all three health outcomes regardless of education, marriage, economic well-being, or social network. We also find three distinct paths linking bullying experiences in childhood to health outcomes in adulthood.

The first path goes through socio-economic attainment. People who were victims of bullying during childhood tend to have lower education and worse standard of living that in turn lead to deteriorating health outcomes compared to those without the experience of bullying. Disadvantaged socio-economic status, especially lower economic well-being, acts as the key mediating factor. The second path pertains to family events, and we considered the role of marriage. Marriages are associated with better health conditions, but individuals with bullying experiences in childhood are less likely to get married than those without such experiences. The lower chances of marriage among bullying victims result in worse health outcomes.

The third path goes through social network ties. The likelihood of having a friend or not turned out to be a crucial factor in the pathway from bullying to health outcomes. People with the experience of bullying at school are more likely to have no friends than those without bully experiences, and the lack of friendship relationships leads to worse health conditions. However, even after taking all these intervening factors together, the indirect paths explain only a small portion of the association between school bullying and health inequalities. This result suggests that there are other possible paths linking bullying experiences in childhood to adult health conditions.

With regard to gender differences, our results suggest virtually no gender differentiation in the extent of the association between school bullying and health outcomes and the decomposition pattern except for one instance where the indirect effect on activity restriction was not significant among women. Overall, the persistent effects of bullying into adulthood and the role of mediating factors in explaining the underlying mechanisms are similar between men and women.

Our findings parallel those of previous research [11–14, 22–24, 26, 60], which reported that the experience of bullying at school had lasting effects on mental and physical health years later. Bullying victimization is associated with deteriorating health outcomes in adulthood. On the other hand, our study shows that the ability of mediating factors to explain the relationship between school bullying and health outcomes is modest. Previous studies have emphasized the significant role played by intervening factors, particularly within the school setting. Chai et al. [11] showed that relationships with parents, teachers, and peers were important mediators, and Varela et al. [13] reported on the importance of the school level socio-economic status. However, our data set did not contain detailed information about the schools attended by the respondents.

Our findings align with those of Zhou and Zhou [40], who indicated that the principal route from childhood bullying experiences to adult mental health is direct. We observed a comparable pattern across three health outcomes, encompassing both mental and physical health. However, while Zhou and Zhou focused on subjective variables as mediating factors, such as satisfaction with children and partners, social activities, and social support, our research instead highlighted objective life-course events, including educational attainment, economic status, marriage, and the presence of social network ties. Our study represents one of the first systematic examinations of the role of objective life-course events in mediating the association between childhood bullying experiences and adult health outcomes. Despite the difference in mediating factors, both studies suggest that these mediators explain only a modest portion of the observed association between childhood bullying victimization and adult health outcomes. Objective life-course events did not significantly outperform subjective satisfaction in this regard. Both factors made modest contributions to explaining the association under investigation. This implies the potential existence of additional mediators not accounted for in these studies.

Sweeting et al. [60] found that negative experiences in childhood, such as bullying and abuse, can lead to later negative life events like serious accidents, illness, and divorce, which, in turn, affect mental and physical health in adulthood. However, it should be noted that Sweeting et al. [60] used the cumulative number of high-level stress events as mediating factors, while our study did not include a range of adverse experiences that happened during adulthood. By excluding these adult experiences as intervening factors, our study may have overestimated the direct association between bullying in childhood and health outcomes in adulthood. As Wolke and Lereya [22] summarized in their review, the children of the victims of school bullying were found to exhibit lower educational attainment, diminished earnings, decreased job retention rates, and worse financial management skills. Our study also indicates that school bullying experiences are associated with low education, low economic well-being, and weaker social network ties. However, these observations contribute to explaining only a small part of the overall association between bullying and health outcomes in our study.

Studies on the long-lasting impacts of ACEs suggest possible psychosocial mechanisms linking the associations between ACEs and health. Bourassa et al. [61] affirmed that psychosocial factors including stressful life events, subjective stress level, negative emotionality, and health behaviors (smoking, physical activity, diet, and alcohol consumption) play an important mediating role. Karatekin and Ahluwalia [62] found that ACEs are associated with higher levels of perceived stress and lower levels of social support that lead to worse mental health scores. These studies suggest that the experience of bullying victimization can lead to adverse impacts on a wide range of economic, social, and psychosocial characteristics that may act as mediating factors. Research into identifying these intervening factors has just begun, highlighting the need for further investigation in the future.

Our study points out that the experience of being bullied as a child is an important risk factor for increasing health inequalities. Given the life-long negative health impacts of bullying, as Arseneault [29] pointed out, while traditional bullying prevention and intervention programs may improve the lives of young victims currently attending school by reducing the likelihood of being bullied, they are unlikely to solve the problems of those who were victimized in the past. There is an imminent need to consider relief systems and policies for past victims of bullying at school. According to Idsoe et al. [63], research on post-traumatic stress disorder (PTSD) symptoms related to school bullying has been limited, and their own study did not reveal PTSD symptoms persisting into adulthood. Further investigation is necessary to understand how PTSD symptoms resulting from bullying victimization at school can be mitigated.

There are several limitations to this study. First, as stated above, we had a modest number of intervening factors that mediate the association between bullying experiences in childhood and health outcomes in adulthood. The inclusion of other possible mediators is most likely to reduce the direct effect of bullying on health outcomes. The JLPS asked questions on a range of work-related factors such as working hours, training opportunities, and workplace conditions. Adverse workplace relationships, especially the presence of an oppressive boss and uncooperative colleagues, for example, may trigger the recollection of bullying incidents in childhood. These variables provide clues about how childhood bullying experiences are ultimately linked to adult health conditions.

Second, we considered bullying experiences at school as an example of ACEs. However, there are other sets of negative experiences, such as parental death, parental separation and divorce, physical and psychological abuse, physical and mental neglect, family substance misuse and mental disorders, and domestic violence. Future research should study the cumulative impacts of a wider range of adverse experiences. Third, because the issue of bullying at school pertains to experience during childhood, there is a possibility of recall bias [7]. People with certain characteristics (such as personality traits) which are related to health outcomes may have better memory than those without those characteristics.

Fourth, this study did not consider the problem of sample attrition of respondents. As the panel survey extends over several years, there is a tendency for some respondents to discontinue their participation in the survey. It is possible that people with health problems are more likely to drop out of the survey. Previous research [64] which examined the factors associated with attrition in the JLPS survey reported that young men and people who had plans to move their residence were more likely to drop out of the survey. However, it did not consider health status as one of the determinants of attrition, so the impact of health status on attrition is unknown. These issues warrant attention in future studies.

## 5. Conclusion

This study suggests that the experience of being bullied at school during childhood has long-lasting impacts on health outcomes in adulthood. The associations between bullying and health outcomes persist regardless of social background and mediating factors of education, marriage, economic well-being, and social networks. Policy measures aimed at not only preventing childhood bullying but also mitigating its adverse consequences in adulthood should be considered to assist individuals who have endured such experiences.

We can draw two significant policy implications from our findings. First, given that bullying can have both immediate and enduring health consequences, it becomes imperative to proactively prevent its occurrence within school environments. The active engagement of teachers and parents is pivotal in mitigating the risk of bullying. The Ministry of Education should articulate a clear definition of bullying and provide comprehensive guidelines for its prevention, ensuring that every school can implement them effectively. Local government, including the board of education, must also actively participate in efforts to mitigate the risks of bullying within schools. In cases of severe bullying incidents, reporting to the police may be necessary. Equipping teachers with training programs to identify early signs of bullying can deter its persistent manifestation. A unified commitment from teachers, counselors, social workers, parents, and students to combat bullying is essential for its eradication from school premises.

Second, support interventions are needed for adults who endured bullying during their youth. Focusing solely on at-risk school children is insufficient. The issue of bullying extends beyond the confines of schools, and it is crucial to acknowledge that its effects can persist beyond school settings. Our results suggest that social network ties play an important mediating role. Broader support networks encompassing family, friends, and counseling resources should thus prove effective. As adults transition from school to work, they typically spend a substantial amount of their social life in the workplace. Although this is not directly addressed in our analysis, workplace conditions and support networks at work hold promise as intervention avenues for adults who endured bullying during their youth.

Finally, it is also important to conduct a follow-up survey targeting children who have encountered bullying at school. Adults who were previously victims of bullying at school require targeted long-term care and support initiatives. By acknowledging and addressing the unique challenges and circumstances faced by adults who suffered from bullying victimization during their formative years, targeted interventions can ensure that support is extended where it is most needed.

## Supporting information

S1 File Data availability statement. (DOCX)

S2 File Ethics approval document. (PDF)

S3 File Ethics statement. (DOCX)

S4 File Financial disclosure statement. (DOCX)
