# Supplementary material for: Bullying experiences in childhood and health outcomes in adulthood
Source: PLoS One. 2024 Jul 15;19(7):e0305005. doi: 10.1371/journal.pone.0305005 (PMC11249246; doi:10.1371/journal.pone.0305005)
Supplement: S1 File — (DOCX) [file pone.0305005.s001.docx]

**Data Availability Statement**

The panel surveys analyzed in the current study are available from the Social Science Japan Data Archive (SSJDA), Center for Social Research and Data Archives, University of Tokyo. To access the datasets, researchers must register at SSJDA Direct (<https://ssjda.iss.u-tokyo.ac.jp/Direct/?lang=eng>). Instructions for utilizing SSJDA Direct can be found at the following URL: <https://csrda.iss.u-tokyo.ac.jp/english/infrastructure/access/apply.html>. The dataset numbers and titles are as follows: "PM140 Japanese Life Course Panel Survey for the Middle-aged (JLPS-M), wave1-14, 2007-2020" and "PY140 Japanese Life Course Panel Survey for the Youth (JLPS-Y), wave1-14, 2007-2020." Researchers are required to integrate the two datasets.
