# Supplementary material for: Bullying experiences in childhood and health outcomes in adulthood
Source: PLoS One. 2024 Jul 15;19(7):e0305005. doi: 10.1371/journal.pone.0305005 (PMC11249246; doi:10.1371/journal.pone.0305005)
Supplement: S2 File — (PDF) [file pone.0305005.s002.pdf]

東京大学社会科学研究所研究倫理審査委員会

審査結果通知書

Notification of Review Results by the Research Ethics Review Committee, Institute of Social Science, The University of Tokyo

令和５年１１月２８日

November 28, 2023

申請者

東京大学社会科学研究所

特別教授石田浩殿

Applicant

Hiroshi Ishida, University Professor,
Institute of Social Science, The University of Tokyo

東京大学社会科学研究所

研究倫理審査委員会委員長

田中隆一

Ryuichi Tanaka,
Chairman, Research Ethics Review Committee,
Institute of Social Science, The University of Tokyo

受付番号：23 12

Reference number

研究課題：格差の連鎖・蓄積モデルからみたライフコースと不平等に関する総合的研究

（継続サンプル）

Research Project: A Comprehensive Study of Life Course and Inequality Using the Framework of Cumulative Advantages and Disadvantages (Continuous Study)

上記研究計画について、委員会で審査の結果、下記の判定を行いましたので、ここに通知します。

We hereby notify you that the committee has reviewed the above research plan and made the following decision.

記

判定<br>Decision<br><br>承認する。<br>Approved
条件または理由<br>Condition or Reason<br><br>〔記載なし〕<br>Not stated
承認番号 ：132<br>Approval No.
