# Supplementary material for: Bullying experiences in childhood and health outcomes in adulthood
Source: PLoS One. 2024 Jul 15;19(7):e0305005. doi: 10.1371/journal.pone.0305005 (PMC11249246; doi:10.1371/journal.pone.0305005)
Supplement: S3 File — (DOCX) [file pone.0305005.s003.docx]

**Ethics Statement**

The Institutional Research Ethics Review Board (the Research Ethics Review Committee) at the Institute of Social Science, University of Tokyo, approved the Japanese Life Course Panel Surveys (JLPS) project. We declare compliance with the ethical practices described in the Code of Conduct for Research at the University of Tokyo.

Respondents received an informed consent form by mail in January 2007 (for the continuous sample) and January 2019 (for the refresh sample), describing the study's objectives and purpose, along with details regarding the confidentiality and anonymity of responses. This information was provided prior to the distribution of the questionnaires. Subsequently, the questionnaires were dispatched by mail to all participants, excluding those who opted out of the survey by responding negatively to the initial mail. Trained surveyors from a reputable survey firm conducted visits to the respondents and collected the completed questionnaires. In the case of the 2007 continuous sample, verbal consents were obtained, and these were duly documented by the surveyors at the time of collecting the completed questionnaires. For the 2019 refresh sample, written consents were procured and collected by trained surveyors. Respondents who did not provide consent were excluded from the sample.
