# Supplementary material for: Bullying experiences in childhood and health outcomes in adulthood
Source: PLoS One. 2024 Jul 15;19(7):e0305005. doi: 10.1371/journal.pone.0305005 (PMC11249246; doi:10.1371/journal.pone.0305005)
Supplement: S4 File — (DOCX) [file pone.0305005.s004.docx]

**Financial Disclosure Statement**

This research was supported by KAKENHI Grant-in-Aid for Specially Promoted Research (Grant Numbers JP25000001 and JP18H05204) and Scientific Research (S) (Grant Numbers JP18103003 and JP22223005) from the Japan Society for the Promotion of Science (JSPS). The research support in conducting the panel surveys was obtained from the Institute of Social Science, University of Tokyo, and The Outsourcing, Inc. Yurie Momose acknowledges the Research Fellowship for Young Scientists (22J10114) from the JSPS. The funders had no role in study design, data collection and analysis, decision to publish, or preparation of the manuscript.
